# Miniature Electromagnetic and Mechanical Resonators for Measurements of Acceleration with the Help of Nitrogen-Vacancy Color Centers

**DOI:** 10.3390/mi16121311

**Published:** 2025-11-23

**Authors:** Marina Rezinkina, Oleg Rezinkin, Fedor Jelezko, Claus Braxmaier

**Affiliations:** 1 Institute for Quantum Optics, Ulm University, Albert–Einstein–Allee 11, 89081 Ulm, Germany; oleg.rezynkin@uni-ulm.de (O.R.); fedor.jelezko@uni-ulm.de (F.J.); claus.braxmaier@uni-ulm.de (C.B.); 2 Theoretical Electrical Technique Department, National Technical University “Kharkiv Polytechnic Institute”, 2 Kyrpychova Str., 61002 Kharkiv, Ukraine; 3 Department of Quantum Metrology, Institute for Quantum Technologies, German Aerospace Center (DLR e.V.), 2022 Wilhem–Runge–Straße 10, 89081 Ulm, Germany

**Keywords:** electromagnetic resonators, mechanical resonators, quantum sensors on nitrogen-vacancy color centers, numerical modeling

## Abstract

Using mathematical and physical modeling, we investigate the influence of the configuration and parameters of miniature electromagnetic and mechanical resonators on their output characteristics. Such electromagnetic resonators are required for the microwave excitation of nitrogen-vacancy color centers, which are used as sensors for various physical quantities, including acceleration, force, and magnetic field induction. The mechanical resonators under consideration are designed for measuring acceleration using nitrogen–vacancy color centers. As a result of these studies, we selected the types of miniature electromagnetic and mechanical resonators that ensure the efficient operation of nitrogen–vacancy color center sensors.

## 1. Introduction

Acceleration is an essential parameter for describing the motion of objects, as it is directly related to the forces that cause their velocity to change. The integration of acceleration levels for moving objects is widely used in navigation equipment to determine speed and position. Sensors for measuring acceleration, or accelerometers, not only measure acceleration but also provide information on the acting force. So, accelerometers can be used to determine forces and mechanical stresses, as well as to measure gravitational interactions. Acceleration measurements in the frequency range from fractions of a hertz to tens of kilohertz and are used in space geodesy [1], gravimetric purposes [2], the determination of orientation and position in medical applications [3], navigation [4], metrology [5], and other applications.

Recently developed optomechanical accelerometers possess several properties that enable their widespread application. These properties include high sensitivity [6], immunity to electromagnetic interference [7], and a wide range of measured acceleration frequencies, from stationary to several hundred hertz [8]. The primary scientific areas of application of inertial and hybrid quantum–inertial sensors can be described as follows [6]: the search for dark matter and dark energy using atomic interferometry, the detection of gravitational waves, and monitoring the Earth’s gravitational field in time and its rotation speed in three dimensions, among others. The most common optomechanical accelerometers are for measuring low frequencies [6], and the natural frequency of optomechanical accelerometers usually does not exceed 10 kHz. However, in some cases, such as vibration testing, launcher acceleration spectrum measurements, and acceleration measurements in space, acceleration measurements at higher natural frequencies of optomechanical sensors are required, up to several tens of kilohertz.

Despite all the advantages, using optomechanical sensors is connected with the appearance of drift during long measurements [8]. Therefore, hybrid quantum optomechanical accelerometers are considered to be more advanced sensors, as the quantum part of such accelerometers can substantially correct a drift [9]. Currently, cold atom interferometers are utilized as the quantum component of such sensors [9]. However, these sensors have several limitations, particularly for space missions, including the need to create a vacuum and operate at low temperatures, which increases their weight, size, and cost.

To leverage the advantages of hybrid quantum optomechanical acceleration sensors and mitigate the disadvantages of currently used quantum sensors on cold atoms, it is possible to use sensors based on nitrogen-vacancy (NV) color centers in diamonds as the quantum component of quantum optomechanical acceleration sensors [10].

An NV center, consisting of a nitrogen atom replacing a carbon atom and a lattice vacancy, is a photoluminescent point defect in a diamond. The operating principle of an NV quantum sensor is based on the fact that changes in physical parameters such as pressure, temperature, magnetic, or electric fields directly affect the spin properties of the NV center, which are then detected using optical methods. Applying acceleration-induced pressure to the diamond lattice leads to a coupling between the orbital and spin dynamics of the NV center. This enables highly accurate measurements of pressure and other physical quantities [11].

The use of NV color centers in diamonds for detecting mechanical processes has been the subject of many studies [11,12]. However, measuring accelerations with their help is relatively new [12] and is not fully developed. The advantages of this method lie in the possibility of significantly miniaturizing these sensors, as well as the absence of the need to create a vacuum and operate at cryogenic temperatures, which simplifies their design and reduces their weight, size, and cost. The advantage of using NV color centers in diamonds to measure acceleration is that the measurements are based on absolute physical constants. Therefore, the drift of acceleration measurements by the optomechanical sensors can be effectively corrected. Compared to optomechanical accelerometers, the disadvantages of acceleration measurements based on NV color centers in diamonds include a higher noise level in the output signal and a comparatively longer measurement period. Thus, the proposed combined quantum optomechanical accelerometers are optimal for measuring rapidly changing accelerations and diminishing drift.

Despite the demonstrated potential of using NV color centers in diamonds to measure accelerations [12], implementing this method requires further research, particularly when utilizing it in combined quantum optomechanical accelerometers.

For measuring accelerations, it is more effective to not use a single NV color center in diamonds, but rather their ensembles, as the sensitivity of the mechanical stress measurement in this case will be significantly higher. The dimensions of the NV color center ensemble correspond to the cross-section of the NV laser beam exciting it. To manipulate the spin states of the NV color centers, laser pumping is used, and this process is controlled by an applied microwave (MW) magnetic field. Therefore, it is essential to create effective resonators—sources that generate MW magnetic fields at the required frequency and magnetic flux density levels.

MW field-forming systems for this application have been described in the literature. Strip lines or omega-like systems [13] have been developed for small volumes of NV color centers. Various planar systems have been elaborated for larger ensemble volumes, including planar two-loop slot resonators [14] and other types [15]. However, their application for large ensembles of NV color centers in diamonds is limited, because the magnetic field becomes weaker outside the field-forming plane of the system and, therefore, more inhomogeneous. So far, several approaches have been proposed to create uniform fields in a relatively big three-dimensional volume, such as the use of dielectric resonators [16], coils [17], and other systems. Dielectric resonators significantly improve the uniformity of the microwave magnetic field. However, due to the very complex development and production technology, difficulty with adjusting their frequency and amplitude characteristics, nonlinearity of dielectric properties in both the frequency and amplitude ranges, temperature instability, and high sensitivity to the influence of lenses and other metal parts, integrating existing resonators into compact devices for industrial applications is rather complicated.

The creation of resonators is a complex process requiring preliminary research. This study focuses on the development of the key components of the proposed combined quantum-optomechanical accelerometer. These components include a mechanical resonator with embedded NV centers, whose displacement under applied acceleration determines its magnitude, and a microwave field-generating system—a resonator used to control the NV centers. Without a preliminary study, it is impossible to know beforehand which parameters and configurations of these resonators will provide the required output characteristics. A mismatch of the electromagnetic resonator causes incomplete energy transfer and signal distortion. The manufacturing and experimental testing of these miniature resonators are associated with significant costs and time. As experience has shown, even minor changes in specific critical parameters can significantly influence the output characteristics of the resonators.

This paper is organized as follows: Section 2 is devoted to the analysis of the characteristics of existing flat electromagnetic resonators for controlling NV color centers and the development of a more advanced resonator configuration, based on the results of the carried-out investigations. Section 3 details the modeling of the mechanical characteristics of miniature mechanical resonators for measuring acceleration, using a Fabry–Pérot cavity and NV color centers in diamonds. Section 4 concludes the paper.

## 2. Electromagnetic Resonators with Submillimeter Parts for Obtaining Microwave Magnetic Fields for Measurements with the Help of NV Color Centers

Microwave electromagnetic fields (EMF) are used to control the spin states of NV color centers. Magnetic fields drive transitions between the spin states of the NV centers. This enables spin manipulation and the utilization of NV centers in sensors for measuring various physical quantities, such as magnetic field induction, temperature, pressure, and acceleration. NV centers in diamonds are manipulated using MW magnetic fields at frequencies close to *f*_0_ = 2.87 GHz, which corresponds to zero-field splitting [18].

Some applications use ensembles of NV centers implanted on a diamond surface. Reducing the degree of decoherence in ensembles of NV centers requires ensuring radial MF uniformity in the plane of the ensembles, which are approximately nano- or micrometers thick [19]. Such systems are typically implemented as planar resonators. To select the parameters of such systems, we compare their output characteristics.

To effectively control NV centers, electromagnetic resonators must provide sufficiently high levels of magnetic flux density in the direction perpendicular to the NV axis and be matched, i.e., have an impedance close to 50 Ohm. Let us consider two common types of planar electromagnetic resonators for controlling NV centers. As will be shown below, the first type provides sufficiently high levels of magnetic flux density but requires additional measures to ensure matching. The second type can be matched, but its magnetic flux density levels are several times lower. Based on an analysis of the characteristics of these resonators, a new combined configuration was selected that ensures both good matching and sufficiently high levels of magnetic flux density.

**Figure 1:**
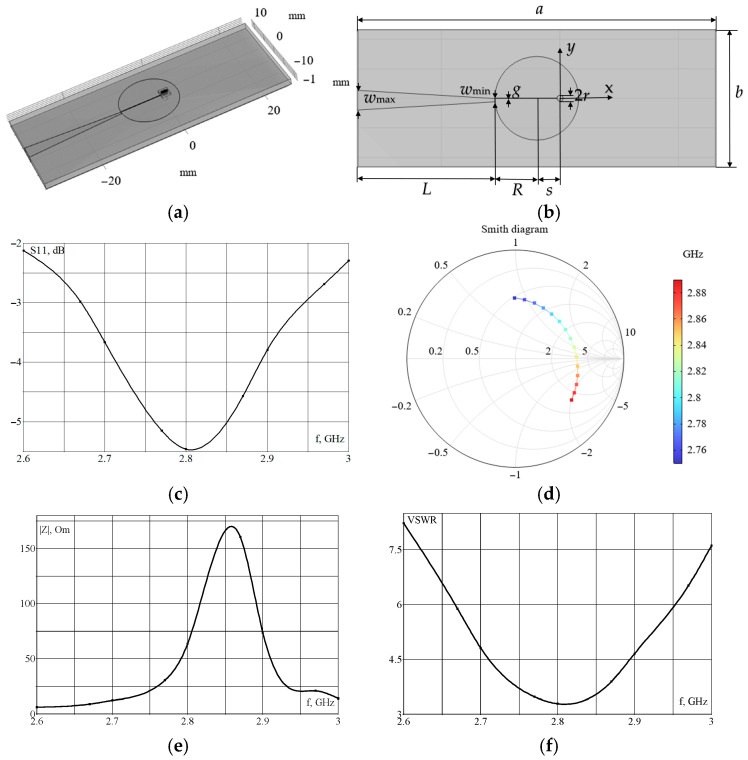

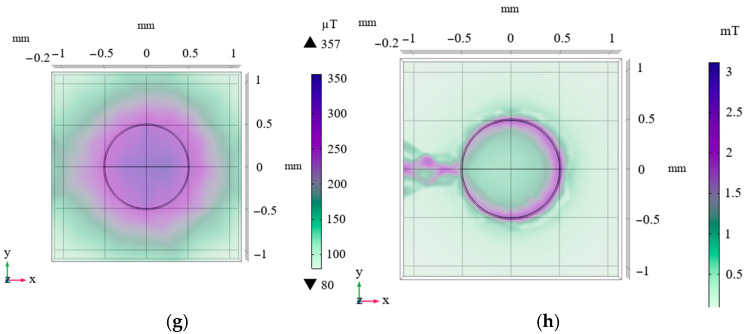
(**a**,**b**) field-forming system under consideration; (**c**) calculated dependence of S11 on frequency; (**d**) Smith diagram; (**e**) dependence of the system impedance module on frequency; (**f**) dependence of VSWR on frequency; (**g**) magnetic flux density around the orifice, with radius, *r*, on the back surface of the diamond at a frequency of 2.87 GHz; (**h**) magnetic flux density around the orifice, with radius, *r*, on the front surface of the diamond at a frequency of 2.87 GHz.

In [15], a flat resonator is described, which enables the achievement of the required magnetic flux density in the NV color centers and a relatively uniform magnetic field distribution. The modeling of electromagnetic processes in this system (see Figure 1a,b) was performed using COMSOL Multiphysics 6.3; we will refer to this resonator as the microwave planar ring antenna from here. The parameters of the simulated system, which includes submillimeter components, are the same as those in [15] and are shown in Figure 1b and Table 1.

Here, *R* is ring radius, *r* is hole radius, *s* is distance between the ring and hole centers, *g* is width of the gap in the ring, *w_d_* is width of the diamond (diamond electrical parameters are as follows: *ε_r_* = 5.68, σ = 0 S/m), *t_d_* 0.51 mm is thickness of the diamond, *t_r_* is thickness of the solder mask (solder mask electrical parameters are as follows: *ε_r_* = 4.3, tan δ = 0.025), *t_a_* is thickness of the metal conductor for the antenna (copper, σ = 5.8 × 10^7^ S/m), and *t_e_* is thickness of the epoxy glass board (FR4, *ε_rb_* = 4.3, tan δ = 0.03). Here, *ε_r_* is the relative permittivity, σ is the conductivity, and tan δ is the loss tangent.

Figure 1c shows the distribution of the parameter S11, the input reflection coefficient. Figure 1 also indicates the modeled distribution of the magnetic flux density at a frequency of 2.87 GHz on the back surface of the diamond, close to the resonator (Figure 1g), and on its front surface (Figure 1h). A comparison of these data (see Figure 1c,g) showed good agreement with the measurement results presented in [15].

However, as shown in the simulation results, this system is not matched, as its impedance differs significantly from the characteristic impedance of 50 Ohm (see Figure 1e). Consequently, a standing wave arises in the circuit. Figure 1f shows the calculated voltage standing wave ratio (VSWR) distribution, which characterizes the impedance mismatch. As can be seen from the dependence (see Figure 1f), the VSWR value is quite significant, even at the resonant frequency of 2.87 GHz. From the simulated Smith diagram (see Figure 1d), it is evident that for this system, in the frequency range under consideration, the matching mode, when the resonator’s impedance is close to 50 Ohm, does not occur.

**Figure 2:**
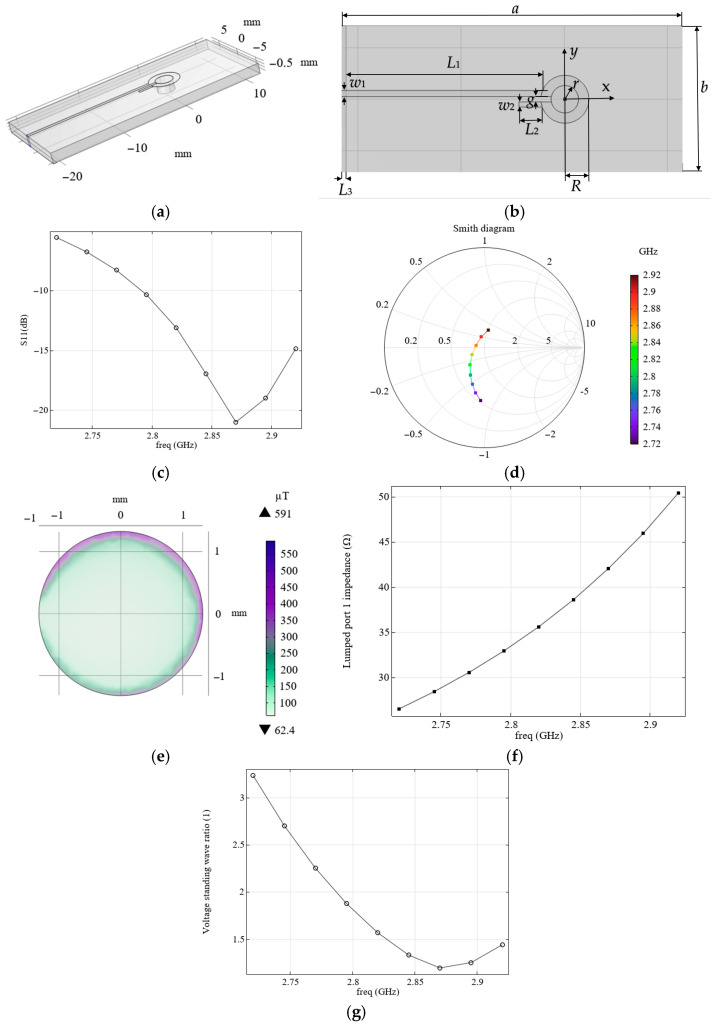
(**a**,**b**) the field-forming system under consideration; (**c**) calculated dependence of S11 on frequency; (**d**) Smith diagram; (**e**) calculated dependence of the magnetic flux density around the orifice with radius, *r*, on the front surface of the diamond, at a frequency of 2.87 GHz; (**f**) calculated dependence of the system impedance module on frequency; and (**g**) calculated dependence VSWR on frequency.

To ensure the matching of the field-forming system with the source, it is proposed to use an alternative configuration, similarly to that described in [20] (see Figure 2a,b, Table 2), which we will refer to as the loop-shaped resonator from here.

With the appropriate choice of parameters, such a field-forming system has an active resistance of approximately 50 Ohm and ensures matching with the source output impedance. The carried modeling of electromagnetic processes showed good agreement with the results presented elsewhere [20,21] (see the calculated distribution of S11 as a function of frequency in Figure 2c).

We have considered two field-forming systems, each with its own advantages and disadvantages. The microwave planar ring antenna produces a magnetic field with a higher magnetic flux density than the loop-shaped resonator (see Figure 1h and Figure 2e). However, the loop-shaped resonator has a match with the source, since its impedance module is close to 50 Hz (see Figure 2f), unlike the microwave planar ring antenna (see Figure 1e). This also follows from the Smith diagram of the loop-shaped resonator (see Figure 2d), which, unlike the Smith diagram for the microwave planar ring antenna (see Figure 1d), passes through the real axis near level 1 that corresponds to the matching regime.

Let us consider the use of an electromagnetic resonator in real-world applications, where there are limitations on its size, due to its proximity to the optical system, the object of study, sources of a constant magnetic field, or other reasons typical of mobile devices. It is also necessary to consider the presence of a microscope near the MW structure, typically with a conductive shell on the objective lens. We consider, first, the case where the electromagnetic resonator has a configuration similar to that described in [15] (see Figure 1a,b).

**Figure 3:**
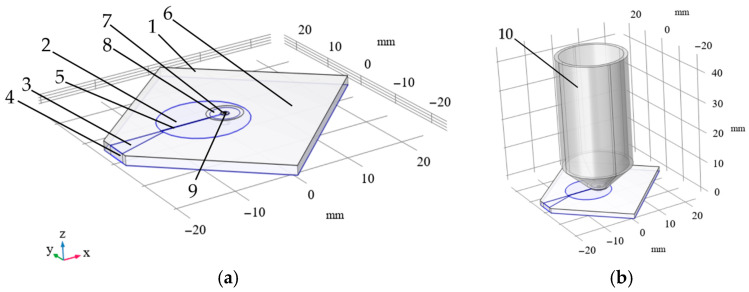
(**a**) Calculation system: 1—board, 2—electrode with radius *R*, 3—feeder, 4—terminals, 5—gap, 6—solder mask; 7—orifice with radius *r*, 8—area without the mask surrounding the orifice, 9—diamond; and (**b**) calculation system with objective lens 10.

Figure 3a shows the system used, which consists of a board, 1, and zones, 2 and 3, covered with metal. Zone 2, having a radius of *R*, is an electrode to which the voltage is supplied through a feeder, 3, from a terminal, 4. A 100 µm-wide gap, 5, is made in the electrode, 2. A mask, 6, is applied to the upper surface of the board, 1, except for an area, 8, surrounding the orifice, 7, into which the diamond, 9, is placed.

**Figure 4:**
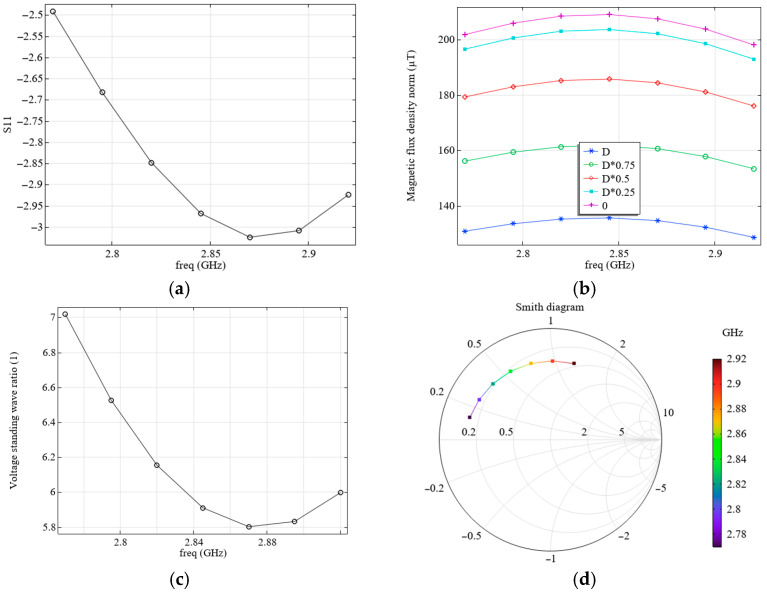
(**a**) Calculated dependence of S11 on frequency; (**b**) calculated dependence of the magnetic flux density on frequency; (**c**) calculated dependence of VSWR on frequency; (**d**) Smith diagram. The calculation was performed at *R* = 7 mm, where the resonator configuration corresponds to Figure 3a without the objective lens.

We analyze the electromagnetic processes in the system with the parameters shown in Figure 3a and Table 3. The simulation results for the S11 parameter (a), and the calculated magnetic flux density levels (b) as a function of frequency (freq), as well as the influence of the distance to the diamond surface (0 corresponds the front surface of the diamond close to the resonator, D, to its outer surface: D is diamond thickness) are shown in Figure 4.

Although the magnetic flux density values shown in Figure 4b are pretty high, in reality, they can be much lower, since these values correspond to the case of supplying all the power to the field-forming system. However, according to the simulation results (see Figure 4a), only a small portion of this energy is supplied to the system under consideration, and most of it is reflected, as this system is not well-matched. It can be seen from the simulation results that the VSWR at the resonance frequency in this case is quite large (see Figure 4c).

**Figure 5:**
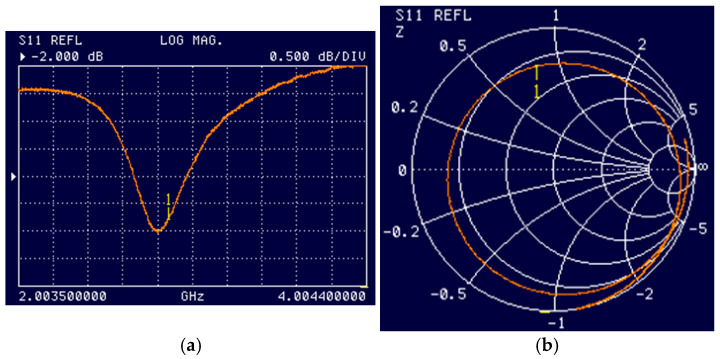
(**a**) Measured dependence of S11 on the source frequency and (**b**) measured Smith diagram. The resonator configuration corresponds to Figure 3a without an objective lens. The “1” mark corresponds to a frequency of 2.87 GHz.

Figure 5 shows the results of experimental studies of electromagnetic processes in this system. A comparison with the measured S11 values (see Figure 4a and Figure 5a) and Smith diagrams (see Figure 4d and Figure 5b) shows good agreement.

**Figure 6:**
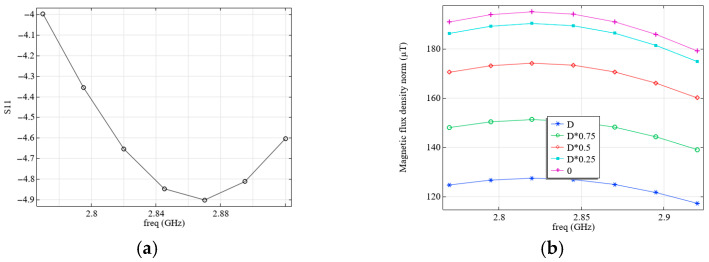
(**a**) Calculated dependence of S11 on frequency and (**b**) calculated dependence of the magnetic flux density on frequency. The calculation was performed at *R* = 7.6 mm, taking into account the presence of a conductive shell on objective lens 10, where the resonator configuration corresponds to Figure 3b.

Under the real operating conditions of the field-forming system under consideration, a microscope with a conductive shell on the objective lens (see Figure 3b) is used for optical excitation and reading the sensor’s NV color centers. The presence of such a conductive object reduces the resonant frequency and the magnetic flux density values. To increase the resonant frequency, the radius of electrode 2 (see Figure 3a) was changed from *R* = 7 mm to *R* = 7.6 mm; all other parameters remained unchanged, as listed in Table 3. The distance of the conductive objective lens from the diamond was set as equal to 600 µm. The results of calculating the values of S11 (a) and the levels of magnetic flux density (b), depending on the frequency and the distance to the diamond surface, are shown in Figure 6.

**Figure 7:**
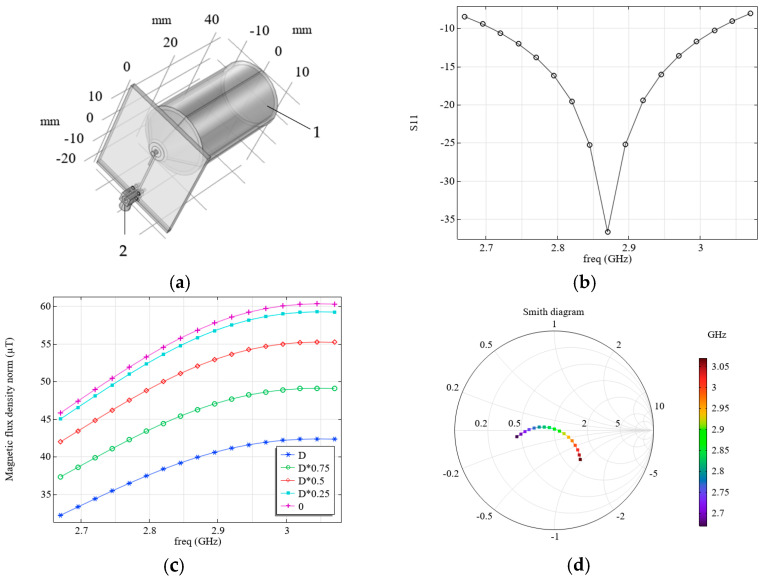
(**a**) Considered field forming system: 1 is the objective lens, 2 is the connector MMCX; (**b**) calculated dependence of S11 on frequency; (**c**) calculated dependence of the magnetic flux density on frequency; and (**d**) Smith diagram.

As follows from the experiment and calculation, the field-forming system considered, which is similar to the one proposed in [15], is mismatched, since its resistance differs significantly from 50 Ohm (see Figure 1e). Thus, despite the relatively high levels of the magnetic flux density occurring in such a system, these values correspond only to the case when special measures are taken to match it with a source. To match the field-forming system, we employed another configuration, similar to that described in [20]. We varied the parameters of the field-forming system in this configuration so that its external dimensions corresponded to the considered practical application and were the same as those of the system in Figure 3a,b. Figure 7 shows the simulation results: S11 (b) and magnetic flux density levels (c), which depend on frequency and distance to the diamond surface. The system configuration corresponds to Figure 2a, and its parameters are presented in Table 4. The presence of an objective lens (see 1 in Figure 7a) and a connector MMCX (see 2 in Figure 7a) were taken into account.

**Figure 8:**
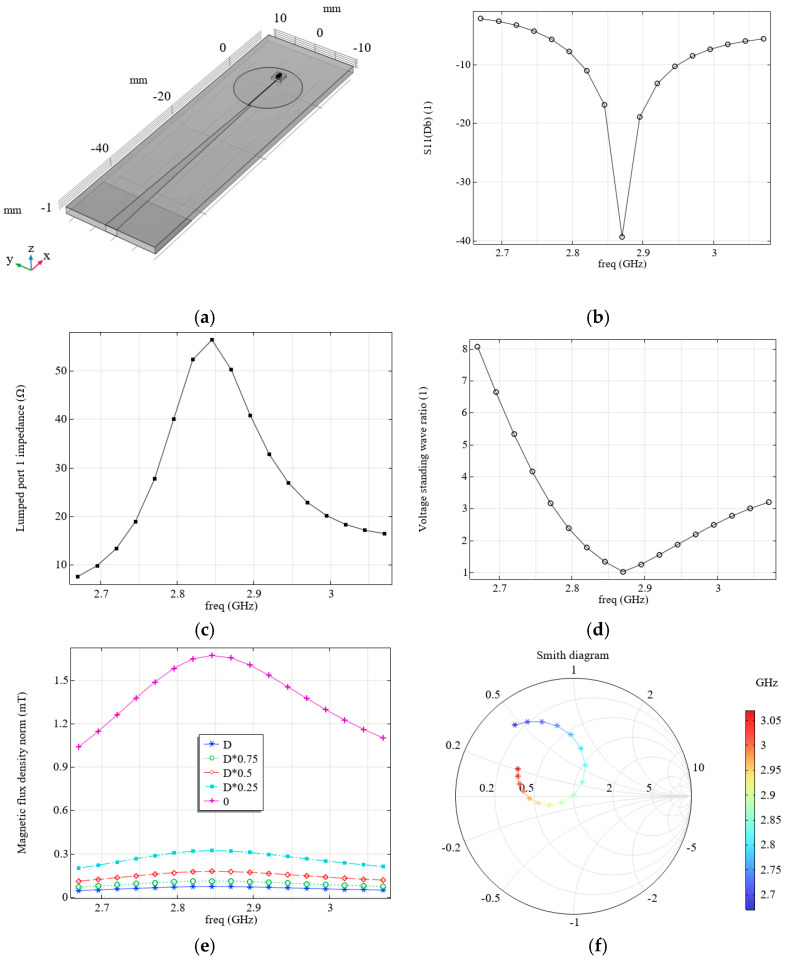
(**a**) Considered field forming system; (**b**) calculated dependence of S11 on frequency; (**c**) calculated dependence of the system impedance module on frequency; (**d**) calculated dependence of VSWR on frequency; (**e**) calculated dependence of the magnetic flux density on frequency; and (**f**) Smith diagram.

We propose a resonator with a new configuration that combines the advantages of the microwave planar ring antenna and the loop-shaped resonator (see Figure 8a). While maintaining approximately the same magnetic flux density levels as the microwave planar ring antenna (see Figure 8e), this resonator has an impedance module close to 50 Ohm (see Figure 8c) and a VSWR value close to 1 (see Figure 8d) at the resonant frequency (see Figure 8b). From the analysis of the output parameters of the resonators considered above, it follows that to improve the matching, part of the metal coating should be removed from the back side of the board. Only a strip (length *L*_3_) should be left. During the modeling process of the resonators under consideration, it was also observed that lengthening the feeder, as well as narrowing it on the side of the connection to the source, leads to an increase in the levels of magnetic flux density. The mentioned features were implemented in the proposed resonator. The parameters of the chosen system are presented in Table 5. Resonance at a frequency of 2.87 GHz, which is required to activate NV color centers, was achieved by varying the length and width of the feeder, the ring radius (R), and the fraction of metal left on the outer side of the board (level of *L*_3_). As can be seen from the modeled results of the electromagnetic processes for this case, changing the configuration and parameters of the resonator results in the Smith diagram for a system passing through the center along the abscissa axis at a frequency of 2.87 GHz (see Figure 8f), which means the resonator is matched.

## 3. Mathematical Modeling of Mechanical Processes in Miniature Mechanical Resonators for Measurements of Acceleration with the Help of NV Color Centers

As noted elsewhere [22], NV color centers in diamonds embedded in mechanical resonators are promising components for determining the parameters of mechanical influences. In such systems, mechanical stresses arising from the deformation of resonator elements cause changes in the interaction of quantum dots with the crystal lattice, which can be detected optically. Quantum dots, such as NV color centers in diamonds, are very effective quantum detectors of different physical processes, including mechanical ones. In such systems, the coherent evolution of electron spins associated with NV color centers in diamonds is found to be related to the motion of a mechanical resonator, such as a cantilever. In [23], negatively charged nitrogen-vacancy centers embedded under the membrane surface are used as nanosensors to study the mechanical properties of a thin membrane. The authors of [23] used confocal microscopy to detect membrane deflection when pressure was applied, measuring the propagation of fluorescence from an individual NV color center in diamond. A micron-sized single-crystal diamond cantilever with a single NV inclusion has been investigated elsewhere [24].

A quantum system based on spin-deformed NV color centers for measuring acceleration has been proposed and implemented [12]. The advantages of such a system over existing accelerometers include low power consumption, small dimensions, a frequency range of up to several kilohertz, and satisfactory sensitivity. The proposed accelerometer [12] features a micron-sized diamond membrane with NV color centers at its center, functioning as a mechanical oscillator fixed on both sides. The spin states of the NV color centers were initialized by laser pumping and controlled by an applied microwave electromagnetic field. As a result of applying acceleration, a deformation of the mechanical oscillator with NV color centers occurs, leading to a change in the intensity and contrast of photoluminescence, which allows for measurements of the magnitude of the applied acceleration.

In the first stage, it was decided to use a simple yet effective design of a mechanical resonator—specifically a rectangular plate—and to analyze its characteristics based on the character of the plate edges’ fastening. Let us consider the design of a mechanical resonator that can be used as a hybrid device, allowing for the simultaneous measurement of acceleration using a Fabry–Pérot cavity and NV color centers in diamonds. Here, a 2000 × 2000 × 50 µm artificial diamond plate is used as a mechanical resonator. The plate has embedded NV color centers located beneath its surface, at a depth of approximately 10 nm. Suppose such a plate is fixed along its contour, linking it to a moving object. In that case, a force will act on the plate, causing the acceleration to be measured, and it will vibrate at the frequency of the applied force and associate it with acceleration (ω). By measuring the magnitude of the displacement *Z* of the plate using a Fabry–Pérot cavity, the acceleration, *a*, with which the entire object moves, can be determined. To ensure a linear dependence of acceleration on the measured displacement, the maximum frequency, ω, of the force, *F*, applied to the object must be an order of magnitude smaller than the first natural frequency of the mechanical resonator used, *f*_0m_ = ω_0m_/2π [6]. It should be noted that since *Z* (ω) ≈ *a (ω*)/ω_0m_, for the same applied *F*, and therefore the same sought-after acceleration *a*, the measured displacement *Z* will decrease significantly with increasing ω_0m_, due to the necessity of the mechanical resonator rigidity increasing. Since measuring tiny displacements is difficult, increasing ω_0m_ above 10∙ω does not seem practical.

Thus, the natural frequency is a crucial characteristic that determines the optimal frequency range for the measured acceleration. The analytical calculation of the natural frequency, which coincides with the first eigenfrequency (*f*_1m_ = *f*_0m_), is only possible for simple mechanical resonators, such as springs. Determining the eigenfrequencies of more complex structures is typically performed using numerical methods. Another essential characteristic of mechanical resonators is the second eigenfrequency (*f*_2m_): it must be significantly greater than the first for the Z (ω) dependence to remain close to linear over the operating frequency range (ω < 0.1∙ω_0m_) [6,8]. Modeling of mechanical processes for eigenfrequencies and displacement field determination was performed using COMSOL Multiphysics 6.3.

**Figure 9:**
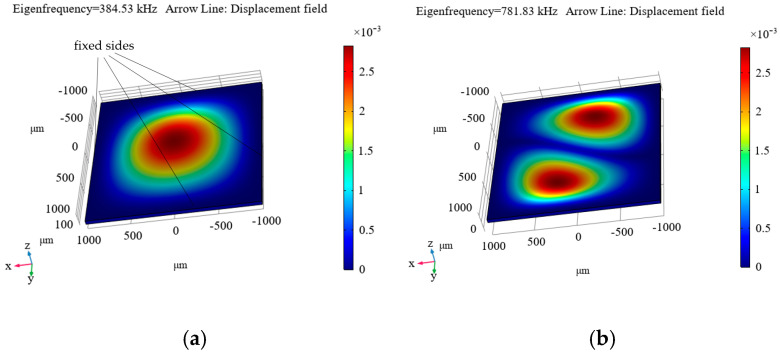
Calculated displacement mode for the first (**a**) and second (**b**) eigenfrequency, for the case of fixing the diamond plate on four sides. *f*_0m_ = *f*_1m_ = 384.53 kHz, *f*_2m_ = 781.83 kHz.

**Figure 10:**
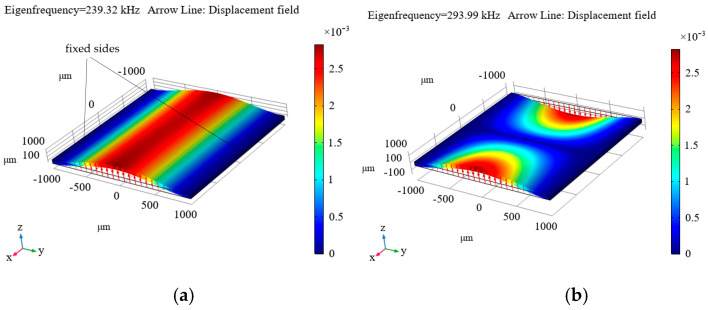
Calculated displacement mode for the first (**a**) and second (**b**) eigenfrequency, for the case of fixing the diamond plate on two sides. *f*_0m_ = *f*_1m_ = 239.32 kHz, *f*_2m_ = 293.99 kHz.

**Figure 11:**
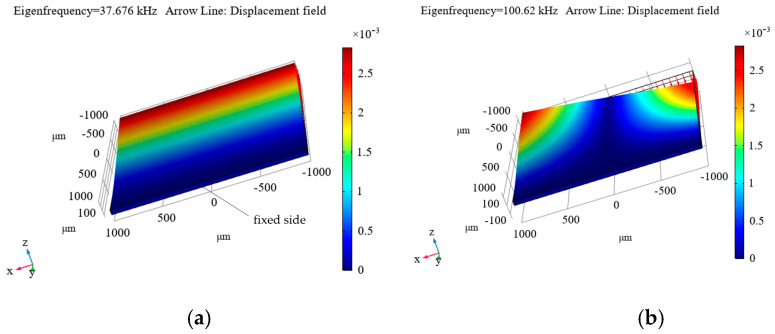
Calculated displacement mode for the first (**a**) and second (**b**) eigenfrequency, for the case of fixing the diamond plate on one side. *f*_0m_ = *f*_1m_ = 37.676 kHz, *f*_2m_ = 100.62 kHz.

Figure 9, Figure 10 and Figure 11 show the simulated distributions of the displacement field at the calculated first (a) and second (b) eigenfrequencies (*f*_1m_ and *f*_2m_ are given in the upper left corner of each distribution and in the captions to them) for the cases of fixing the diamond plate on four sides (Figure 9), on two sides (Figure 10), and on one side (Figure 11).

As can be seen from these distributions, the most suitable option is to clamp the plate on one side (see Figure 11), as this results in the first eigenfrequency of 37.676 kHz, which is not too high, but is still sufficient for measuring accelerations in the frequency range up to 4 kHz, which suffices for the maximum necessary frequency level of the measured accelerations. For the other options, the level is significantly higher, necessitating the measurement of tiny displacements, which is a rather complicated process. Additionally, a resonator with the plate clamped on one side ensures a sufficiently small ratio of the first to the second eigenfrequency (see Figure 11).

The advantage of this mechanical resonator configuration lies in its ability to simultaneously record the applied acceleration using a Fabry–Pérot cavity (for details, see [6,8]) and optically read the photoluminescence of NV color centers in diamonds. Such a plate can be placed inside the elaborated electromagnetic resonator, provided it has the appropriate dimensions.

Thus, this design of a mechanical resonator is suitable for a combined accelerometer in which the acceleration measurement is performed by both optomechanics and a Fabry–Pérot cavity, as well as by NV color centers.

## 4. Conclusions

Using numeric simulation, the output characteristics of two common types of resonators were investigated. These resonators include the microwave planar ring antenna [15] (see Figure 1) and the loop-shaped resonator [20] (see Figure 2). It has been shown that resonators of the first type provide relatively high levels of magnetic flux density, but they are mismatched. Resonators of the second type can be matched with an appropriate choice of parameters. Still, the magnetic flux density levels in them are significantly lower than in resonators of the first type. A comparison of the frequency dependences of the input reflection coefficient, S11, and the Smith diagram obtained from modeling and measurements for these resonator configurations showed good agreement. The models tested in this manner were used to select the resonator parameters, taking into account the size limitations that are inherent in real applications, as well as the presence of conductive components, such as the objective lens and connector, for example, MMCX (see Figure 3, Figure 6 and Figure 7).

A new configuration of an electromagnetic resonator is proposed, which can be used to excite NV centers in diamonds in quantum acceleration sensors, allowing for its matching in the loop-shaped resonator, and obtaining magnetic flux density levels no less than those in the microwave planar ring antenna (see Figure 8).

Modeling of processes in mechanical resonators that can be used in hybrid quantum acceleration sensors using a Fabry–Pérot cavity and NV centers in diamonds has been performed. It was shown that the most suitable configuration is the diamond plate fixed on one side (see Figure 11).

Work on using newly designed MW and mechanical resonators in sensors with NV color centers in diamonds is in progress.

## Figures and Tables

**Table 1:** Main parameters of the microwave planar ring antenna (configuration is shown in Figure 1a,b), *ε_rb_* = 4.3.

Parameter	*a*	*b*	*L*	*s*	*w*_max_	*w*_min_	*g*	*R*	*r*	*t_r_*	*t_a_*	*t_e_*	*w_d_*
Value [mm]	60	23	23	3.9	3.3	0.54	0.1	7	0.5	0.03	0.018	1.6	0.5

**Table 2:** Main parameters of the loop-shaped resonator (configuration is shown in Figure 2b), *ε_rb_* = 4.4.

Parameter	*a*	*b*	*L*_1_	*L*_2_	*L*_3_	*w*_1_	*w*_2_	*G*	*R*	*r*	*t_r_*	*t_a_*	*t_e_*	*w_d_*
Value [mm]	33	14.2	19.27	2.1	0.5	0.58	0.5	0.508	3.66	1.32	0	0.021	1.6	0.5

where *L*_3_ is the length of the metallization on the back side of the board (see Figure 2b).

**Table 3:** Main parameters of the microwave planar ring antenna (configuration is shown in Figure 1b and Figure 3a), *ε_rb_* = 4.

Parameter	*a*	*b*	*L*	*s*	*w*_max_	*w*_min_	*g*	*R*	*r*	*t_r_*	*t_a_*	*t_e_*	*w_d_*
Value [mm]	36	32	4.6	3.9	3.5	0.58	0.1	7	0.75	0	0.018	1.5	0.5

**Table 4:** Main parameters of the loop–shaped resonator (configuration is shown in Figure 2b and Figure 7a), *ε_rb_* = 3.6.

Parameter	*a*	*b*	*L*_1_	*L*_2_	*L*_3_	*w*_1_	*w*_2_	*g*	*R*	*r*	*t_r_*	*t_a_*	*t_e_*	*w_d_*
Value [mm]	36	32	19.27	5	0.5	0.58	0.5	0.1	1.8	0.75	0	0.05	1.52	0.5

**Table 5:** Main parameters of the optimized electromagnetic resonator (configuration is shown in Figure 8a, parameter names are shown in Figure 1b and Figure 2b), *ε_rb_* = 4.5.

Parameter	*a*	*b*	*L*_1_	*L*_3_	*w*_max_	*w*_min_	*g*	*R*	*r*	*t_r_*	*t_a_*	*t_e_*	*w_d_*
Value [mm]	71.8	30.75	45	11.97	2.6	0.54	0.1	10.25	0.75	0	0.018	1.5	0.5

## Data Availability

The data presented in this study are available upon request from the corresponding author.
